# Supplementary figures and images for: Development of a bedside score to predict dengue severity
Source: BMC Infect Dis. 2021 May 24;21:470. doi: 10.1186/s12879-021-06146-z (PMC8142072; doi:10.1186/s12879-021-06146-z)

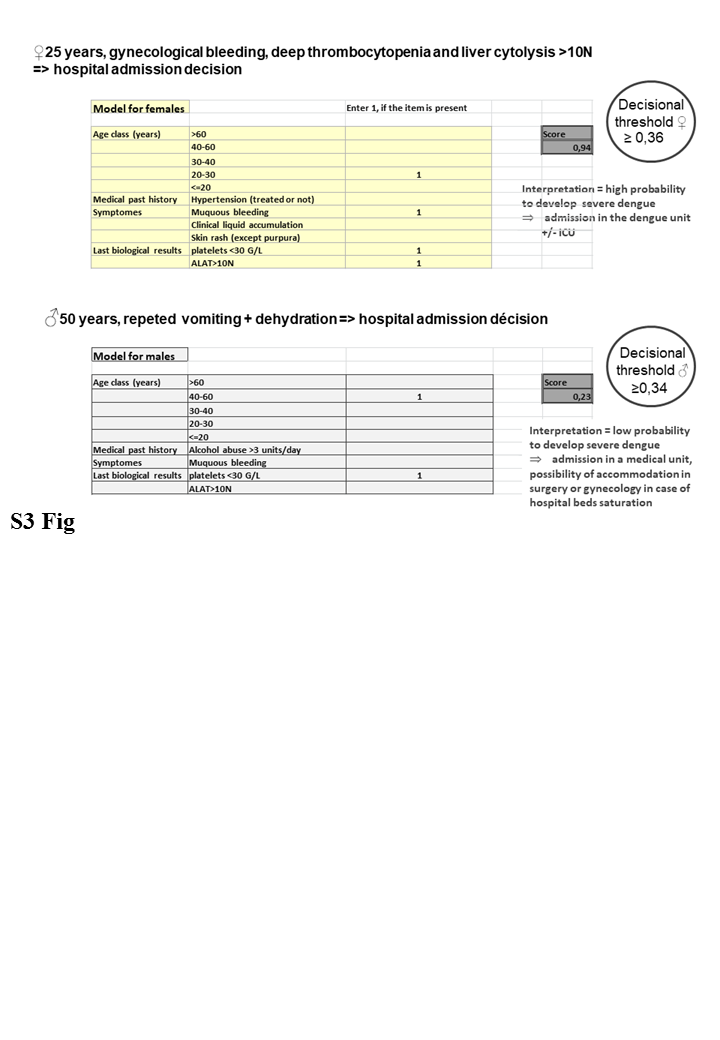

Supplement: Supplementary file 3 — Additional file 3: S3 Fig. Examples of scoring to estimate the risk to develop severe dengue using data available at the moment of hospital admission decision. In the upper example, the score of the female patient is above 0.36, indicating a high risk to develop severe dengue. In the lower example, the score of the male patient is below 0.34, indicating a low probability to develop severe dengue. [file 12879_2021_6146_MOESM3_ESM.tif]

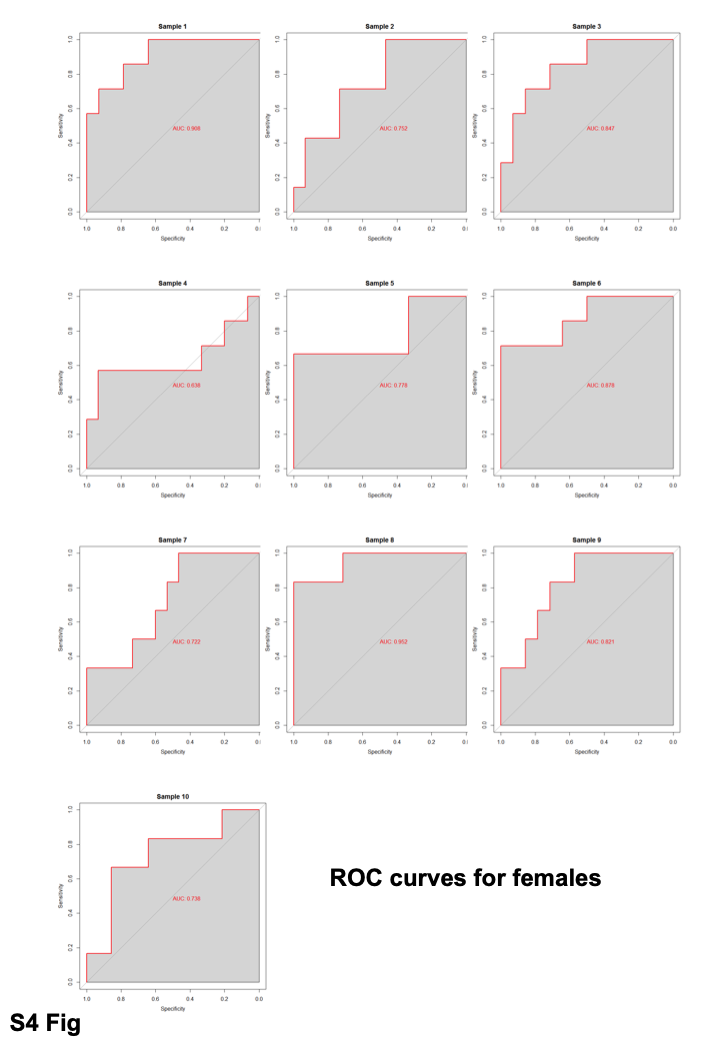

Supplement: Supplementary file 4 — Additional file 4: S4 Fig. Receiving operating curves obtained for females in the k-fold cross-validation procedure. [file 12879_2021_6146_MOESM4_ESM.tiff]

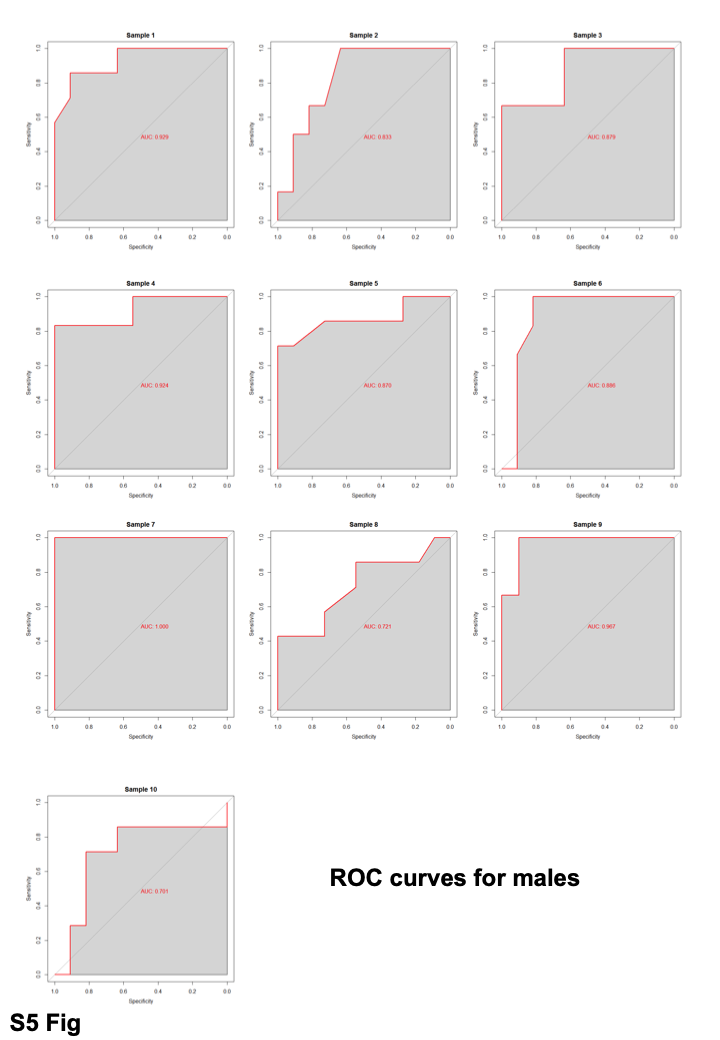

Supplement: Supplementary file 5 — Additional file 5: S5 Fig. Receiving operating curves obtained for males in the k-fold cross-validation procedure. [file 12879_2021_6146_MOESM5_ESM.tiff]
