# Supplementary material for: Development of a bedside score to predict dengue severity
Source: BMC Infect Dis. 2021 May 24;21:470. doi: 10.1186/s12879-021-06146-z (PMC8142072; doi:10.1186/s12879-021-06146-z)
Supplement: Supplementary file 6 — Additional file 6: S6 Fig. Contingency tables showing the performance of the models for the prediction of dengue severity on dengue 2018 outbreak in New Caledonia. Absolute numbers of severe dengue observed in the dataset and predicted by the models are shown for the model for females (upper table) and the model for males (lower table). [file 12879_2021_6146_MOESM6_ESM.docx]

**Supplementary table S6 Fig**

**Contingency tables showing the performance of the models for the prediction of dengue severity on dengue 2018 outbreak in New Caledonia**

Females (n=66)

| Classification of the patients  Result of the model | Severe dengue | Non-severe dengue |
| --- | --- | --- |
| Severe dengue | 11 | 6 |
| Non-severe dengue | 4 | 45 |

Males (n=64)

| Classification of the patients  Result of the model | Severe dengue | Non-severe dengue |
| --- | --- | --- |
| Severe dengue | 16 | 13 |
| Non-severe dengue | 3 | 32 |
